# Effect of contact vector direction on achieving cavotricuspid isthmus block

**DOI:** 10.1038/s41598-023-29738-y

**Published:** 2023-02-13

**Authors:** Hitoshi Sumi, Tadashi Hoshiyama, Kenji Morihisa, Katsuo Noda, Shozo Kaneko, Hisanori Kanazawa, Masanobu Ishii, Koichiro Fujisue, Daisuke Sueta, Seiji Takashio, Hiroki Usuku, Kenichi Matsushita, Kenichi Tsujita

**Affiliations:** 1 Department of Cardiovascular Medicine, Graduate School of Medical Sciences, Kumamoto University, 1-1-1, Honjo Chuo-Ku, Kumamoto, 860-8556 Japan; 2 Kumamoto Chuo Hospital, 1-5-1, Tainoshima Minami-Ku, Kumamoto, 862-0965 Japan

**Keywords:** Cardiac device therapy, Interventional cardiology

## Abstract

Cavotricuspid isthmus (CTI) ablation is an important treatment strategy for CTI-dependent atrial flutter (AFL). The location of the catheter contact area is confirmed by the contact vector direction (CVD) through three-dimensional mapping during the procedure. However, the relationship between CVD during radiofrequency ablation and its efficacy in achieving CTI block has not been clarified. This study aimed to investigate the relationship between CVD and efficacy in achieving CTI block. CVDs during radiofrequency ablation were divided into proximal vectors against the distal tip (P-vector) and other vectors (normal-vector). In 39 patients who underwent CTI linear ablation, the CTIs were divided into two segments: the tricuspid valve area (anterior) and inferior vena cava area (posterior). The frequency of the residual conduction gap was compared between segments in which the P- and normal-vectors were observed. P-vectors were observed in 13 of the 78 segments. The median ablation index was not significantly different between segments in which the P-vector and normal-vector were observed (398.2 [384.2–402.2] vs. 393.3 [378.3–400.1], *p* = 0.15). However, residual conduction gaps were significantly more frequently observed in the segment in which the P-vector was observed than those in which only the normal-vector was observed (6/13, 46.2% vs. 3/65, 4.6%; *p* < 0.01). During a 6-month follow-up, two patients required a second session of ablation due to AFL recurrence. A residual conduction gap was observed in one patient at the site where the P-vector was observed in the first session. Avoiding the P-vector might be an important factor in improving CTI block and reducing AFL recurrence.

## Introduction

Radiofrequency catheter ablation (RFCA) is the first-line therapy for cavotricuspid isthmus (CTI) dependent atrial flutter (AFL). It is usually performed to create a line of block from the tricuspid annulus to the inferior vena cava using an ablation catheter. In addition, with respect to arrhythmia recurrence, it has been shown that RFCA shows better results than antiarrhythmic agents’ therapy^1,2^. During the procedure, the output, ablation duration, and contact force affect lesion formation. Therefore, the ablation index (AI), which combines output, ablation duration, and contact force, is used as a degree of lesion formation parameter^3,4^. However, even when sufficient AI is achieved at each ablation point, an incomplete conduction block can emerge in some cases^3,4^. Once an incomplete conduction block is created, an additional ablation point or a subsequent line is required. Moreover, another atrial flutter might emerge owing to the additional ablation points^5^. This incomplete conduction block might be due to catheter movement during respiratory cycles^6^ and the anatomical complexity of the right atrium^7–9^. Although the issue of catheter movement due to respiration cycles has been overcome using deep sedation, to overcome insufficient catheter contact and stability against the tissue due to the anatomical complexity represented by pouch and eustachian ridge remains challenging. Techniques for CTI anatomy evaluation during the procedure, namely angiography or intracardiac echocardiography (ICE), are effective but somewhat troublesome^10,11^, because angiographic imaging is not a real-time evaluation of the catheter-CTI contact situation, and ICE images tend to be obscure, especially in the proximal area where the eustachian ridge is observed. Therefore, to overcome this problem, we focused on other parameters, particularly the contact vector direction (CVD). Because, it has been shown that the catheter CVD represents the catheter-target tissue contact situation during pulmonary vein isolation^12^. In addition, even with the same AI value, the size of lesion has been varied depending on the vector^12^. Simply put, if the contact vector is proximal against the distal tip of the catheter (P-vector), the lesion size is significantly smaller than that in other vector^12^. Because it has been shown that the anatomical structure is more complex in CTI than in the pulmonary vein vicinity^13^, CVD might be more important in CTI than the vicinity of the pulmonary vein with respect to lesion formation. Therefore, we investigated the relationship between the CVD and the completion of the CTI block.

## Methods

### Study population

The study population comprised 39 consecutive patients who underwent their first CTI linear ablation using radiofrequency ablation at Kumamoto Chuo Hospital between February 2019 and November 2019. The primary arrhythmias for catheter ablation were CTI-dependent AFL (17 patients), uncommon AFL (3 patients), and persistent atrial fibrillation (19 patients).

Treatment with an antiarrhythmic drug was discontinued for at least five half-lives prior to the ablation procedure. Before the ablation procedure, oral anticoagulation was maintained for at least 1 month without interruption of the warfarin therapy. Treatment with dabigatran, rivaroxaban, apixaban, and edoxaban was omitted only on the morning of the procedure. The study was approved by the [Human Research Committee of the Kumamoto Chuo Hospital] and [Human Research Committee of the Kumamoto University Hospital]. These committees are the national ETHICS Committees. Written informed consent was obtained from all patients. All methods were performed in accordance with the relevant guidelines and regulations.

### Cardiac catheterization

A 6-French duo-decapolar steerable catheter (BeeAT, Japan Lifeline, Tokyo, Japan; or EP star, Japan Lifeline, Tokyo, Japan) was percutaneously inserted with the tip positioned in the coronary sinus via the right jugular vein. Shape and electrode disposition are identical in both BeeAT and EP star catheters, except for cardioversion function included in BeeAT catheter. Therefore, BeeAT was used for the patients who needed cardioversion during the procedure. Subsequently, a 6-French duo-decapolar catheter (Inquiry, Abbott, Chicago, IL, USA) was percutaneously inserted into the right atrium and placed around the tricuspid annulus via the right femoral vein. Electroanatomical mapping was performed using a multipolar mapping catheter (PENTARAY, Biosense Webster, Inc., Diamond Bar, CA, USA). This electroanatomical mapping was merged with right atrial 3D-CT performed prior to the procedure. Prior to catheter ablation, intravenous heparin was administered to maintain an activated clotting time of 300 to 350 s.

### CTI linear ablation

CTI linear ablation was performed by integrating the right atrial images using an irrigation catheter (ThermoCool Smart Touch SF catheter; Biosense Webster Inc., Diamond Bar, CA, USA). Radiofrequency energy was delivered at 35 W. Ablation was guided using an automated annotation system (VISITAG module; Biosense Webster Inc., Diamond Bar, CA, USA). The parameters were set as follows: (1) catheter stability range of motion, < 3 mm; (2) catheter stability duration, > 3 s; (3) contact force, > 3 g, time, > 25%; (4) tag size, 4 mm in diameter. At the beginning of this study, the target AI for CTI ablation was unknown; therefore, we adopted a target AI value of 400 following that of atrial fibrillation a type of atrial arrhythmia^14^. The initial CTI ablation line was created using a single line. To evaluate the conduction gap location, additional ablation was not performed before the initial CTI ablation line was created. A successful CTI block was defined as follows: first, the right atrial activation sequence of the duodecapolar catheter (Inquiry; Abbott) positioned around the tricuspid annulus was counterclockwise (medial to lateral) during CS ostium pacing; second, differential pacing was observed^1,2^. If the initial ablation line did not create a conduction block, an additional ablation was performed to complete the block.

### Analysis of CVD and residual conduction gap

**Figure 1:**
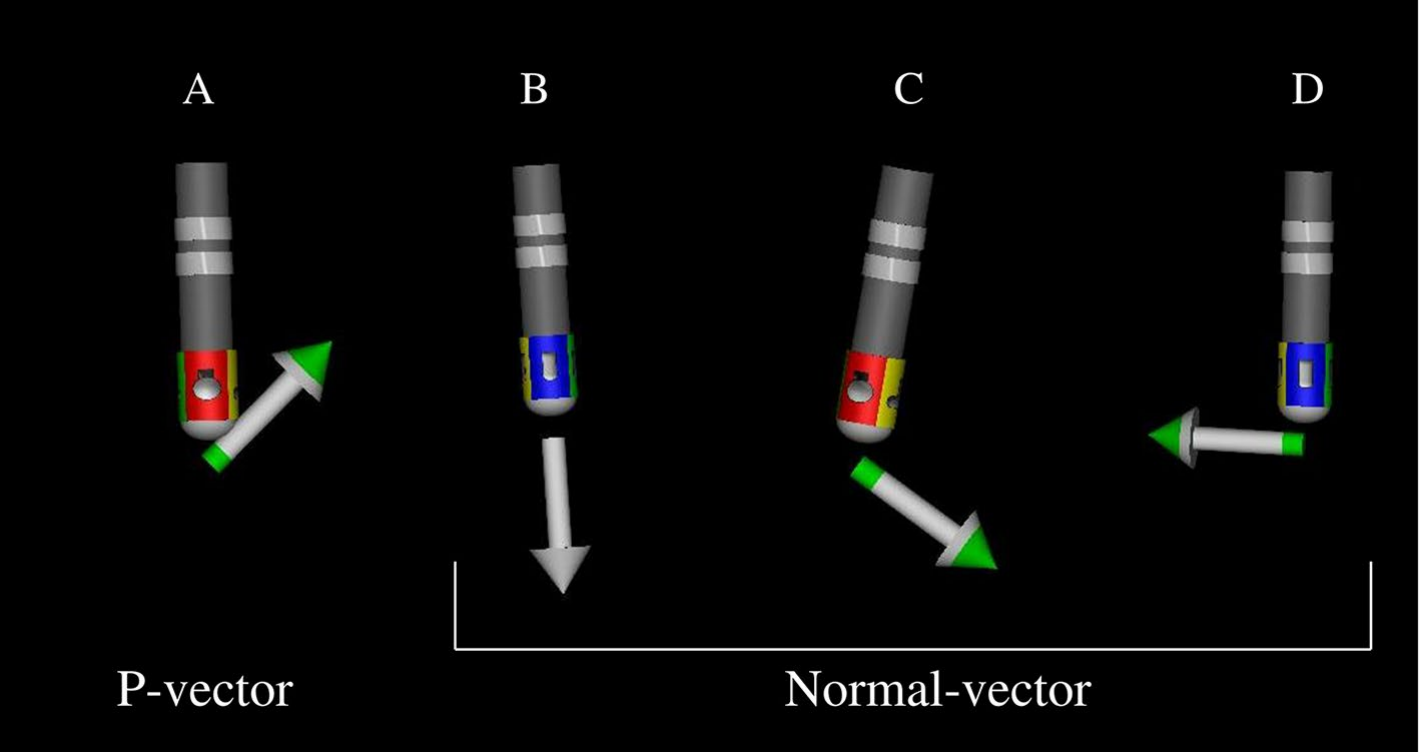
Type of contact vector direction against the distal tip of the catheter: proximal vector (**A**), straight vector (**B**), diagonal vector (**C**), and vertical vector (**D**).

The CVD of the ablation point was analyzed using the CARTOREPLAY module (Biosense Webster, Irvine, CA, USA) after the procedure. This module provides information about the catheter throughout the entire period of the target procedure, including the catheter location, electrocardiograms, and catheter contact vector^12^. CVD during radiofrequency ablation was evaluated in the stable expiratory phase. With respect to mechanism of CVD representation, the distal tip electrode of the ablation catheter was connected to a small-precision spring located between the second and third electrodes. The deflection of this spring enables not only the precise calculation of force in grams, but also the estimation of CVD^12^. Figure 1 shows the types of CVDs, namely, the proximal vector (Fig. 1A), straight vector (Fig. 1B), diagonal vector (Fig. 1C), and vertical vector (Fig. 1D) against the distal tip of the catheter. It has been shown that if the contact vector is proximal (P-vector), the lesion size was significantly smaller than that in other vectors^12^. This is because, during the P-vector, the distal tip of the catheter was not touched but only proximal tip was touched^12^. Therefore, we divided the CVD into a P-vector and other vectors (normal-vector), according to a previous report^12^.

**Figure 2:**
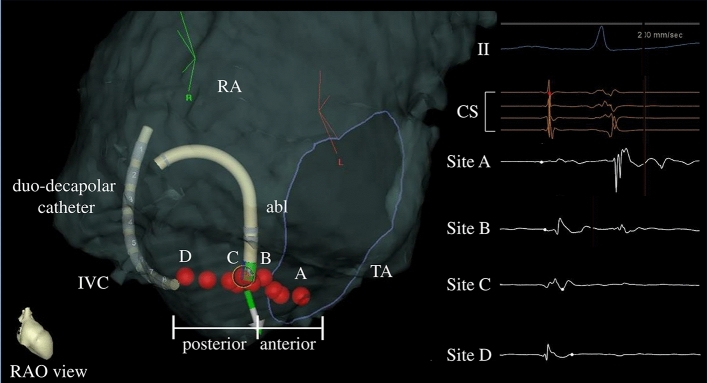
Ablation points of CTI and the corresponding local potentials. The anterior segment defines the area in which atrial and ventricular potentials were observed. The posterior segment defines the area in which only atrial potential was observed. Abl, ablation catheter; CTI, cavotricuspid isthmus; IVC, inferior vena cava; RA, right atrium; TA, tricuspid annulus.

Each CTI was divided into two segments. Figure 2 shows the segments dividing the CTI, namely, the tricuspid valve area (anterior segment), and inferior vena cava area (posterior segment), according to the local electrograms. The anterior segment was defined as the area where atrial and ventricular potentials were observed. The posterior segment was defined as the area in which only the atrial potential was observed. Furthermore, the segments in which the P-vector was observed were distinguished from those in which only the normal-vector was detected. The number of the residual conduction gap was analyzed for each segment.

### Follow-up

Physical examination and surface electrocardiogram (ECG) were performed at 1, 3, and 6 months after the procedure at the outpatient clinic for all 39 patients. Furthermore, 24-h Holter ECG was performed at 3 and 6 months. Recurrence was defined as the presence of AFL or atrial fibrillation on surface ECG or 24-h Holter ECG for > 30 s. A second session was performed when arrhythmia recurrence was observed. In these cases, CTI conduction was evaluated, and the relationship between the residual conduction gap and the P-vector during the first session was analyzed.

### Statistical analysis

All continuous data were skew distributed by the Shapiro-Wilk test; therefore, the continuous data were expressed as medians (interquartile range) and analyzed using non-parametric tests. Continuous data include age, body mass index, e-GFR, BNP, left ventricular ejection fraction (EF), and AI. Categorical variables include P-vectors, residual conduction gap, and recurrence. For comparison among these categorical data, Fisher’s exact test was used due to the small number of events. AI, frequency of residual conduction gap, and recurrence were compared between the patients who observed the P-vector and those who observed only normal-vector during the procedure. A *p*-value <0.05 denoted a statistically significant difference between the two groups. All statistical analyses were performed using the R software (R Foundation for Statistical Computing, Vienna, Austria).

## Results

### Patient characteristics

Table 1 summarizes the patients’ characteristics. A total of 28 men and 11 women with a median age of 68.0 [63.5–73.5] years were enrolled. Nineteen patients had hypertension, and seven patients had diabetes mellitus. Seven patients had a history of heart failure secondary to arrhythmia. The median left ventricular ejection fraction measured by echocardiography was 64.0 [57.0–68.0] %. With respect to additional ablation, pulmonary vein isolation was performed in 31 patients, superior vena cava isolation was performed in 27 patients, box isolation was performed in 5 patients, and non-PV foci ablation was performed in 5 patients due to atrial fibrillation. Mitral isthmus ablation was performed in 2 patients, chemical ablation of the marshal vein in 1 patient, right atrial lateral wall linear ablation in 1 patient, and superior vena cava isolation in 1 patient due to non-CTI-dependent AFL. No major procedural complications were noted. The analyzed ablation points in this present study were 355 points in 78 segments during for all 39 patients. Nine residual conduction gaps were observed in 9 patients (23.1%) out of 39 patients after the initial ablation was completed. However, CTI block were successfully achieved following residual conduction gap ablation. No cases were finished the procedure in unsuccessful despite numerous ablation points or procedure time.

**Table 1:** Patient characteristics.

N	39
Age, year	68.0 [63.5–73.5]
Male gender, n (%)	28 (71.8%)
BMI, kg/m^2^	23.7 [21.7–25.7]
eGFR, ml/min/1.73m^2^	62.8 [51.5–69.0]
BNP, pg/ml	67.1 [29.9–110.0]
EF, %	64.0[57.0–68.0]
HT, n (%)	19 (48.8%)
DM, n (%)	7 (17.9%)
HF, n (%)	7 (17.9%)
Procedural characteristics	
PV isolation, n (%)	31 (79.5%)
SVC isolation, n (%)	28 (71.8%)
Box isolation, n (%)	5 (12.8%)
Non PV foci ablation, n (%)	5 (12.8%)
Mitral isthmus ablation, n (%)	2 (5.1%)
RA lateral wall ablation, n (%)	1 (2.5%)
Chemical ablation to marshal vein, n (%)	1 (2.5%)

BMI, body mass index; DM, diabetes mellitus; EF, ejection fraction; HF, heart failure; HT, hypertension; PV, pulmonary vein; RA, right atrium; SVC, superior vena cava.

### Percentage of segments in which the P-vector was observed

The P-vector was observed in 13 of 78 segments. Five (12.8%) were in the anterior and eight (20.5%) were in the posterior segments, and there was no significant difference between these segments (*p* = 0.56).

### Comparison of median AI at each segment

Table 2 summarizes the median AI at each segment, as well as the median AI at segments in which the P-vector and normal-vector were observed. As indicated by these data, the median AI did not significantly differ between all segments in which the P-vector and normal-vector were observed (398.2 [384.2–402.2] vs. 393.3 [378.3–400.1], *p* = 0.15). In addition, regarding each anterior and posterior segments, the median AI was not significantly different between the segment in which the P-vector and normal-vector were observed at the anterior segments (400.7 [398.5–401.4] vs. 400.8 [393.1–406.6], *p* = 0.49) and posterior segments (401.7 [394.7–403.0] vs. 390.5 [365.8–399.1], *p* = 0.07). All segments, except for the posterior segments in which only the normal-vector was observed, met the target AI value (> 400).

**Table 2:** The median AI at each segment.

	Total segments	P-vector segments	Normal-vector segments	*p*-value
All segments	395.9 [381.8–400.9]	398.2 [384.2–402.2]	393.3 [378.3–400.1]	0.15
Anterior segments	400.8 [394.0–403.5]	400.7 [398.5–401.4]	400.8 [393.1–406.6]	0.49
Posterior segments	393.8 [374.5–401.2]	401.7 [394.7–403.0]	390.5 [365.8–399.1]	0.07

### Frequency of residual conduction gap

**Figure 3:**
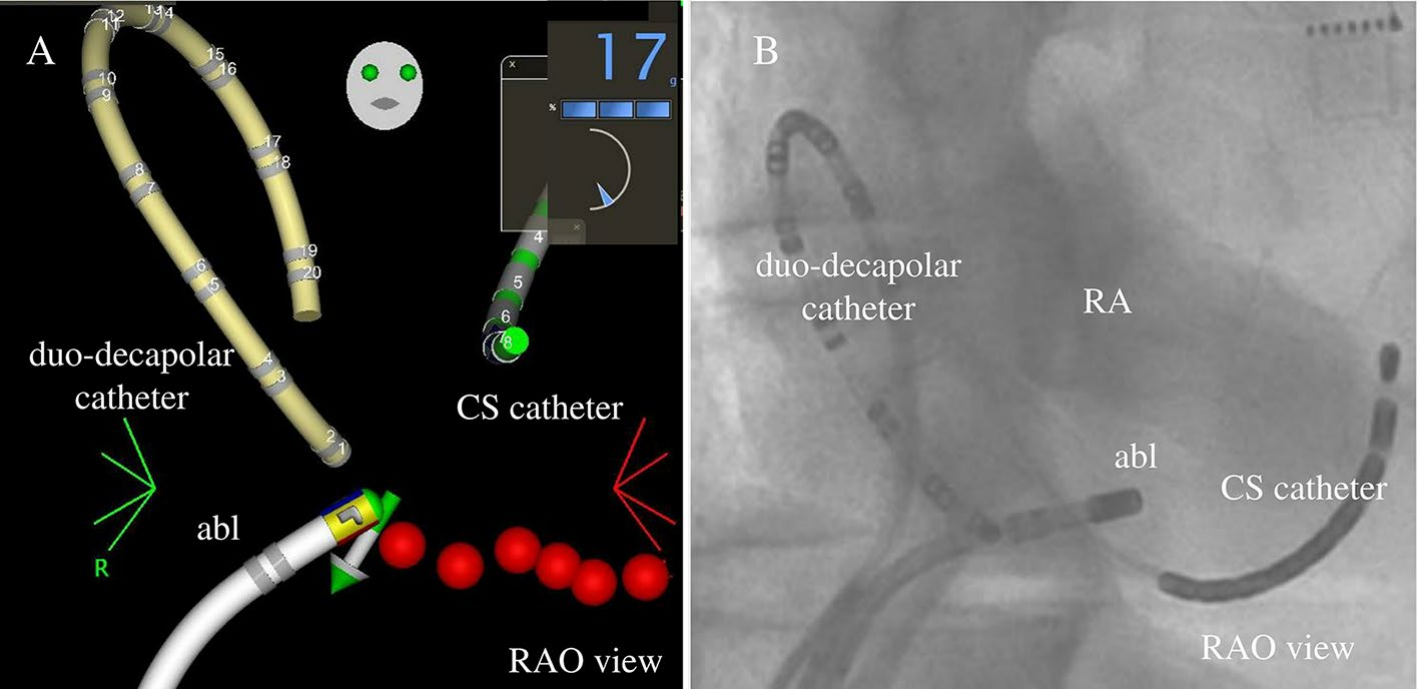
(Representative figure): 3-D mapping image (panel **A**) and corresponding fluoroscopic image (panel **B**) for the P-vector at the CTI posterior segment. Abl, ablation catheter; CTI, cavotricuspid isthmus; CS, coronary sinus.

Table 3 lists the frequencies of the residual conduction gap at each segment. In the 13 segments in which the P-vector was observed, six residual conduction gaps were detected (46.2%). In comparison, in the remaining 65 segments in which only the normal-vector was observed, three residual conduction gaps were noted (4.6%). The residual conduction gap was significantly more frequently discerned in segments in which the P-vector was observed than in those in which only the normal-vector was observed (*p* < 0.01). Additionally, a significant difference was observed between the anterior (*p* = 0.01) and posterior (*p* = 0.02) segments. Figure 3 shows the 3-D mapping image and the corresponding fluoroscopic image of the P-vector at the posterior segment of the CTI. As shown in Fig. 3A, the catheter contacted the posterior segment on 3D mapping with the P-vector at 17 g. However, the distal tip did not touch the CTI and only the proximal edge was touched on fluoroscopic image (Fig. 3B).

**Table 3:** The frequency of residual conduction gap at each segment.

	Residual conduction gap			p-value
	Total segments	P-vector segments	Normal-vector segments	
	78	13 (16.7%)	65 (83.3%)	
All segments	9/78 (11.5%)	6/13 (46.2%)	3/65 (4.6%)	< 0.01
Anterior segments	2/39 (5.1%)	2/5 (40.0%)	0/34 (0%)	0.01
Posterior segments	7/39 (17.9%)	4/8 (50.0%)	3/31 (9.7%)	0.02

### Follow-up

During the 6-month follow-up, a second procedure was performed in 3 of the 39 patients. CTI-dependent AFL recurrence has also been observed in two patients. Atrial fibrillation recurrence was observed in one patient. The CTI reconnection was evaluated for each patient. Among the three patients, the P-vector was observed only in one patient. Although a residual conduction gap was not observed in the patients who had undergone a second catheter ablation due to atrial fibrillation recurrence, a residual conduction gap was observed in the other two patients.

**Figure 4:**
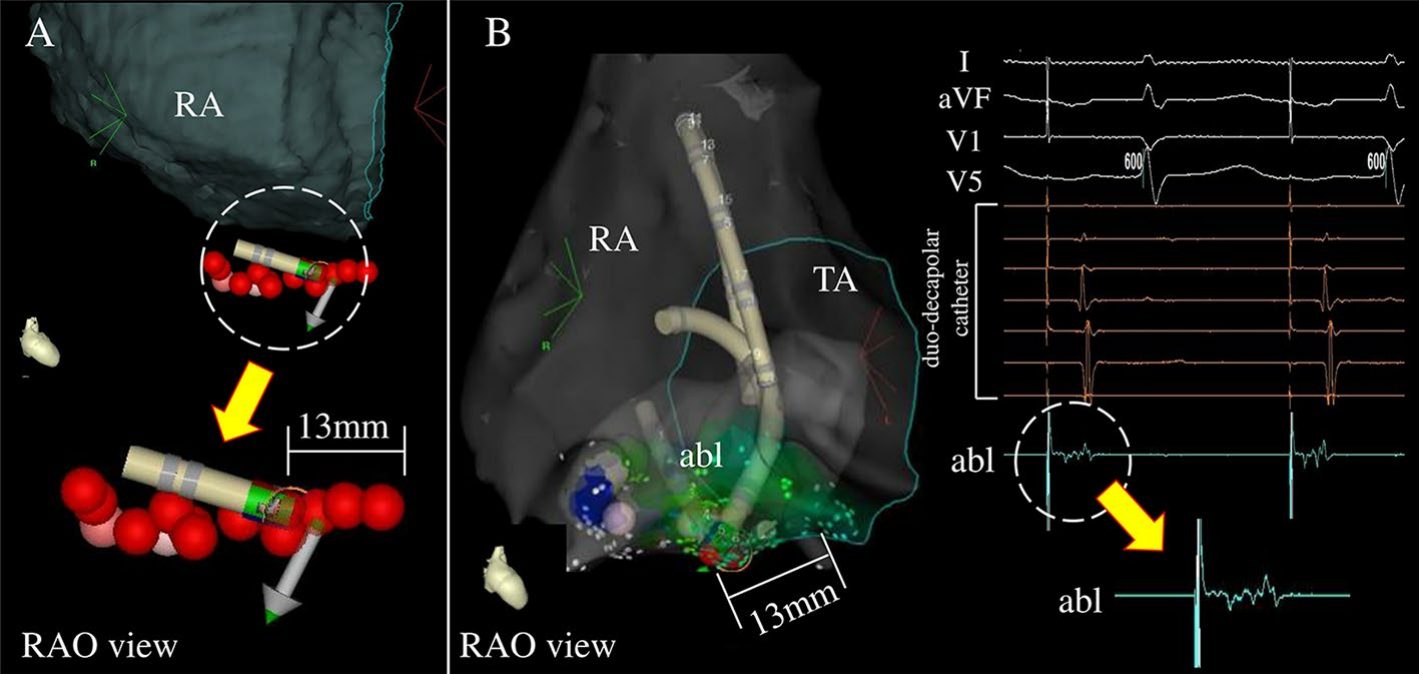
Vector in which the residual conduction gap was observed at first session (panel **A**) and the location and the corresponding local electrogram in which the residual conduction gap was observed at second session (panel **B**). Abl, ablation catheter; RA, right atrium; TA, tricuspid annulus.

Figure 4 shows the 3D mapping image of the first session (Fig. 4A) and the 3D mapping image and local intracardiac electrograms of the second session (Fig. 4B) for the patient with AFL recurrence. As shown in this figure, the CVD indicates the P-vector of the posterior segment in the first session. The diameter of the tricuspid annulus to the site where the P-vector was observed to be 13 mm (Fig. 4A). The electrogram at the corresponding site where the P-vector was observed in the first session represents the fractionated potential (Fig. 4B) in the second session. Radiofrequency catheter ablation at this site immediately completed the CTI block procedure.

## Discussion

### Main findings

The present study yielded the following three major results: first, there was no significant difference in the frequency of the P-vector between the anterior and posterior segments. Second, significantly more residual conduction gaps were detected in segments in which the P-vector was observed than in those in which only the normal-vector was observed. This result was observed in both the anterior and posterior segments of the CTI. Third, although in only one patient, a residual conduction gap emerged during a 6-month follow-up at the site where the P-vector was observed in the first session. A discussion of each result is described below.

### Tendency of the P-vector and anatomical specificity on CTI

In this study, the P-vector was observed in both the anterior and posterior segments of the CTI.

With respect to the relationship between the P-vector and anatomical specificity, we have previously reported the relationship between the P-vector and the anatomical location of the pulmonary vein vicinity^12^. In that report, the P-vector was frequently observed only at the inferoanterior and bottom segments of the right pulmonary vein and superoanterior and inferoanterior segments of the left pulmonary vein. However, the P-vector was rarely observed in other segments^12^. In contrast, the P-vector was observed at both the anterior and posterior segments of the CTI in the present study. This phenomenon might emerge because of anatomical specificity. In fact, it has been shown that the anatomical structure of the CTI is more complex than that of the pulmonary vein, as represented by the eustachian ridge and pouch^9,11^. These structures may cause the creation of a P-vector. Actually, although an appropriate gram was observed in 3D mapping, the fluoroscopic image proved that the distal tip of the catheter was not touched but floated owing to the eustachian ridge (Fig. 3) in the present study. Therefore, in terms of effective lesion creation in radiofrequency ablation, catheter CVD may be more important than the vicinity of the pulmonary vein.

### Residual conduction gap and the recurrence of AFL

In the present study, a CTI block was eventually achieved in all patients; 76.3% of initial lines achieved a successful CTI block. It has previously been shown that AI-guided linear ablation of the CTI results in better outcomes than conventional contact-force-guided ablation, owing to a reduction in the residual conduction gap^3^. However, the completion of a CTI block in initial line ablation greatly varied in other reports, from 50 to 90%^3,4^. This proportion was consistent with the results of the present study. Angiographic imaging and ICE use during the procedure have been reported to be effective in overcoming of this varied range of CTI block completion^10,11^. However, angiographic imaging is not a real-time evaluation of the catheter-CTI contact situation, and ICE images tend to be obscure, especially in the proximal area where the eustachian ridge is observed. In this study, the residual conduction gap was closely associated with the segment in which the P-vector was observed. We have previously reported the relationship between CVD and lesion formation in an experimental model^12^. In that report, although the catheter’s distal tip touches the myocardium in a normal-vector, the catheter’s distal tip did not touch the myocardium but floated in the P-vector. In addition, lesions created by the P-vector were significantly smaller than those created by the normal-vector^12^. This phenomenon is consistent with the results of the present study. Because of the anatomical complexities, such as the eustachian ridge and the pouch in CTI, P-vectors are considered to be observed. Therefore, if the CVD represents the P-vector, poor contact between the catheter distal tip and myocardium might result in the lesions smaller than those of the normal-vector. This might be one of the mechanisms of the emerging residual conduction gap, despite sufficient AI being delivered. To validate this phenomenon, additional experiments or observations using ICE may be needed.

### Residual conduction gap in long-term follow-up

In this study, AFL recurrence was observed in two out of the 39 patients. In one case, the site where the P-vector was observed in the first session represented the residual conduction gap in the second session. In the other case, a residual conduction gap was observed at the site where the residual conduction gap was observed in the first session, however, the CVD was a normal-vector in the first session. This phenomenon was observed in only two patients, suggesting that a residual conduction gap might be one of the factors of AFL recurrence during a long-term follow-up period. Therefore, avoiding the P-vector might be important not only for the creation of a CTI block in the acute phase, but also to avoid AFL recurrence. As a result, it may help in preventing thromboembolic events and heart failure, which represent complications of AFL.

### Study limitations

In the present study, we targeted an AI of 400 at each ablation point. Contrary to the present study, the target AI in CTI ablation tends to be higher. Therefore, if the target AI is higher, the completion of the CTI block may be higher.

Further, we analyzed the relationship between P-vector and gender, and we found P-vectors in 6 out of 56 segments in males (10.7%) and 7 out of 22 segments (31.8%) in females, and there was a significant difference between them (*p* = 0.04). Regarding patient’s characteristics, height and body weight in males were significantly higher than those of females (height: 170.2 [166.3.0–173.3] cm vs. 155.5 [152.9–156.7] cm, *p* < 0.01), (body weight: 69.5 [64.9–77.7] kg vs. 54.9 [47.3–61.5] kg, *p* = 0.04). Therefore, it is unclear whether gender differences affect this result. Although propensity score match analysis may help further analysis, the number of affected patients was not sufficient to carry out the analysis.

## Conclusion

The residual conduction gap was detected significantly more frequently in segments in which the P-vector was observed. This phenomenon might be due to the fact that the lesions created by radiofrequency ablation in the P-vector were significantly smaller than those created in the normal-vector. Therefore, avoiding the proximal vector direction might be an important factor in improving CTI ablation.

## Data Availability

The datasets used and analyzed during the current study available from the corresponding author on reasonable request.
